# Role of Particle Shape on Ground Responses to a Circular Tunnel Excavation in Sandy Soil: Consequences from DEM Simulations

**DOI:** 10.3390/ma15207088

**Published:** 2022-10-12

**Authors:** Zixin Zhang, Xiaogeng Gao, Shuaifeng Wang

**Affiliations:** 1 Department of Geotechnical Engineering, College of Civil Engineering, Tongji University, Shanghai 200092, China; 2 Key Laboratory of Geotechnical and Underground Engineering, Ministry of Education, Tongji University, Shanghai 200092, China

**Keywords:** tunnel excavation, ground response, sandy soil, particle shape, numerical simulation

## Abstract

Due to the sensitivity of sandy soil’s mechanical behavior to the particle shape, it is thus of importance for interpreting the effect of particle shape on the ground response induced by tunnel excavation in sandy formations. We conducted a series of 2D DEM (discrete element method) simulations on a common circular tunnel excavation in sandy soil with variable-shaped particles, which are characterized as two descriptors, i.e., aspect ratio (AR) and convexity (C). The macroscopic responses and the microscopic characteristics of the sandy ground are elaborated in detail. The simulation results show obvious asymmetrical features of the excavated ground, which results from the ground heterogeneity caused by the irregular particle shape. In addition, we investigate the roles of AR and C on the ground response and find that reducing AR or increasing C will enlarge the ground settlement, i.e., the sandy ground deformation is more sensitive to the particles with more irregular shapes. However, elongated particles are beneficial for the generation of soil arching with stronger bearing capacity and thus reduce the soil pressure on the tunnel lining. Our findings have important implications for the safety assessment of the tunnel excavation, as well as other underground structure construction in sandy formations.

## 1. Introduction

Sandy soil is a common kind of granular material widely distributed in the nature, which has the characteristics of large particle size range, complex shape and low cohesion. As typical particle material, its characterizing mechanical properties are generally discontinuity, heterogeneity and anisotropy [1,2,3,4]. The particles in natural sandy soil have various shapes due to grain formation in the depositional environments during the complex geological process [1,4]. The particle shape has been proven to be an important factor affecting the micro and macro mechanical behaviors of sandy soil [4,5,6,7,8,9,10,11] through laboratory experiments and numerical simulations.

The experimental approaches are capable of investigating the macro mechanical behaviors of various types of geomaterials. Based on laboratory triaxial tests, Pan et al. [12] found that the elastic module of granular materials increased with the particle roughness due to enlarged interlocking friction. Igwe et al. [13] investigated the influence of grading distribution on the mechanical properties of silica sands using ring shear tests, demonstrating that the higher the uniformity coefficient is, the higher the shear strength will be. Tutumluer and Pan [14] explored the role of the shape of coarse particles on the strength and deformation of mixture materials using triaxial tests, revealing that the convexity makes a great contribution to the structural strength and stability of the particle-formed mixture under the higher confining pressure. Tsomokos and Georgiannou [15] conducted a series of undrained shear tests on sand samples with same grading but different angularities of grains, showing that the specimens consisting of angular grains exhibited a stable response with increasing strength with strain, while rounded sands of similar density had unstable behavior and reduction in the shear stress after a transient peak strength. Xiao et al. [16] investigated the relationship between the relative breakage index and mechanical indexes using a series of large-scale triaxial tests on Tacheng rockfill material, showing that the fractal dimension of the tested material at the end of the tests was linearly correlated with the void ratio. Laboratory experiments also showed that the critical state friction angle was weakly dependent on the grading [17] while highly related to the content of angular particles [18,19]. Keramatikerman and Chegenizadeh [7] performed a series of static triaxial compression tests on three types of sands and pointed out that increasing the irregularity of particle shape enlarged the critical friction angle. In the tests, it is also found that sand samples with higher irregularity tended to be liquefied later. In addition to the natural sand particles, 3D-printed soil was also used to study the influence of particle shape on the shear behaviors of granular materials [20]. Their laboratory investigations showed that the higher the initial void ratio is, the lower the shear strength will be.

However, the above approaches are unable to capture the microscopic characteristics inside the geomaterials experimentally. Therefore, the discrete element method (DEM) has been developed to be a complementary tool to study particle-formed geomaterials such as sandy soil [21,22]. Xu et al. [23] performed a series of DEM simulations on the drained and undrained triaxial and true triaxial tests and found that the shear strength and the dilation of the granular material increased with increasing irregularity and elongation, which agreed well with the laboratory observation. The DEM simulations on cyclic shear tests showed that the shear modulus and damping ratio can be increased and decreased, respectively, by decreasing the sphericity of particles [24]. For the simulations of direct shear tests, it was found that the particle roughness influences the shear strength by affecting the interparticle friction while the particle angularity impacts the shear strength by affecting the dilation [25]. Tsigginos and Zeghal [26] adopted soil samples of non-spherical particles to analyze the micromechanical effects of particle shape on the low-strain stiffness of granular soils and showed that the low-strain shear modulus of a given granular soil behaves as a function of average particle sphericity and roundness. Lai et al. [9] developed a novel Fourier series-based DEM to capture the particle packing profile, contact fabric and stress–strain evolution for multiple irregular-shaped particle systems. Nguyen et al. [10] generated ellipsoid and cluster particles with different degrees of eccentricity in their DEM simulations, showing that the strain hardening rate of ellipsoids was slightly higher than that of spheres, while the rate was much higher for clustered particles, i.e., more irregular particles show much more dilative tendency compared to spheres and ellipsoids. The particle shape effect on the small strain properties of granular soils was also studied by DEM simulations adopting realistic shape clumps [27]. The shear stiffness of granular soils was found to be positively dependent on the proportion of irregular particles to the sample. Zhu et al. [28] established a cohesionless mixed soil model containing both fine and coarse particles to investigate the effect of coarse particle shape on the shear behaviors of this mixed soil. The roundness and the rolling resistance of coarse particles played different roles on the shear strengths of cohesionless mixed soils.

Concerning tunnelling in sandy formations, numerous studies have been conducted on the ground response induced by excavation, including theoretical analysis [29,30], laboratory model tests [31,32], empirical methods [33,34] and numerical simulations [35,36]. The mechanical properties of sand and the geometrical configuration of excavation are the primary factors dominating the ground response. Accordingly, due to the dependency of the mechanical properties of sandy soil on the particle shape, it is important to interpret the role of particle shape on the ground responses induced by the tunnel excavation [36]. However, only a few studies have been attempted to figure out this crucial issue. Yin et al. [36] built a series of numerical models containing three types of particles (spherical, elongated and tetrahedral particles) to investigate the particle shape effects on the progressive failure of a circular tunnel face as well as the ground surface deformation and the supporting force on the excavation face. A similar issue is the ground response in the trapdoor problem in granular soils formed by irregular particles [37,38], where the particle shape has been proven to play a vital role in the development of soil arching. The consequences from the models containing four types of particles revealed that the final critical height after the trapdoor moving downward increases with decreasing sphericity or increasing roundness, and the enhanced interlocking due to the irregular particles decreases the localization of the shear strain but increases the dilation in shear bands propagating from the edge of the trapdoor [37]. Based on the experimental and DEM-based numerical observations, Ali et al. [38] pointed out that the particle shape has a more significant effect on the formation of soil arching than the particle size. However, up to now, the micro and macro characteristics of ground responses on the cross section due to tunnel excavation have not been well studied in preceding publications, which, thus, motivates the development of our current work.

In this paper, we build a four-particle model to reproduce the mechanical behavior of a type of natural quartz sand with different particle shapes, which are characterized as two descriptors, i.e., aspect ratio (AR) and convexity (C). A series of 2D DEM simulations is conducted on a common circular tunnel excavation in sandy soil to investigate the role of particle shape on the ground response. We analyze the stress states before and after the excavation, the excavation-induced ground settlement and the soil pressure on the tunnel lining from a macro perspective. Afterwards, the microscopic characteristics of the ground response, including particle rotation, stress variation angle, void ratio, coordination number, force chains and shear-slip contact distribution, are demonstrated in detail. Finally, the influences of the particle shape descriptors on the ground response in sandy soil are discussed with respect to the micro and macro perspectives.

## 2. Numerical Model Setup

### 2.1. Generation of Particle Clump

**Figure 1:**
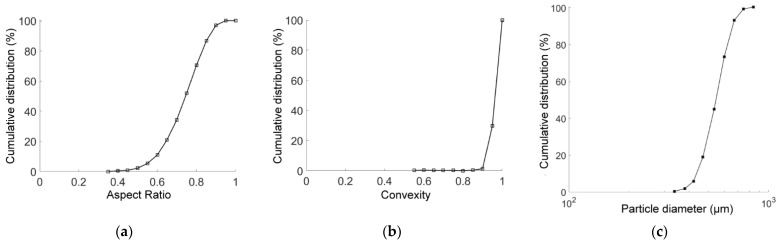
Cumulative distribution curves for (**a**) aspect ratio, (**b**) convexity and (**c**) grading of Fujian sand [39].

**Figure 2:**
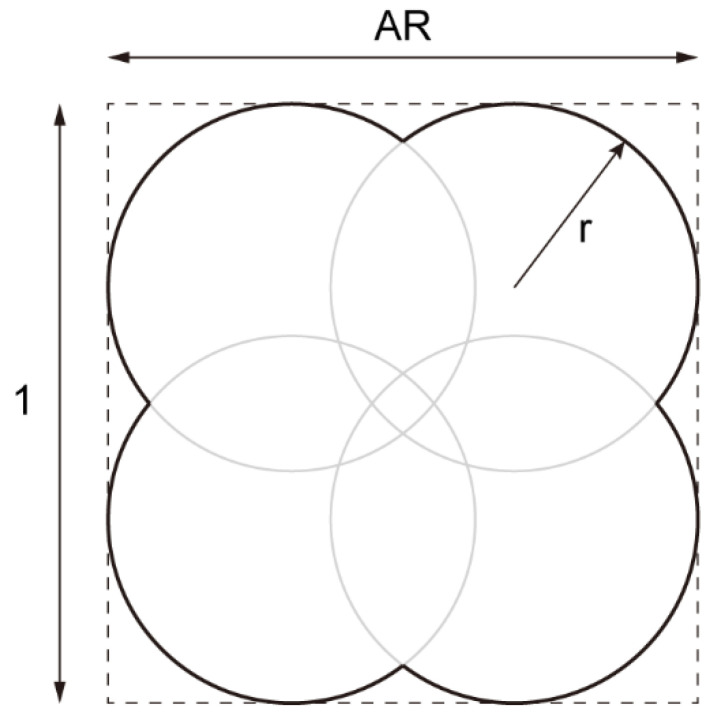
Schematic of the four-particle model to form a particle clump.

In order to comprehensively describe the irregular particle shape and reveal the influence of the particle shape on the macroscopic mechanical properties of the sandy soil, we used two widely used quantitative characterization indicators for shape description: the aspect ratio (AR) and the convexity (C). AR is defined as the ratio of the minimum distance between the parallel tangent lines with respect to a particle’s outer contour to the maximum one, and C is defined as the ratio of the area of concave regions to the particle area. Considering the anisotropy of the realistic particle shape, we adopted the Chinese standard sand (a type of natural quartz sand and usually called Fujian sand) [39] as a calibration reference, of which the statistical results of AR and C are shown in Figure 1a and Figure 1b, respectively. We built a four-particle model to generate a particle clump, where the particles were distributed in a rectangular arrangement, as shown in Figure 2. To obtain the desirable values of AR and C, the particle clump was constructed using the following steps: (1) generate a rectangle (dashed line) with a length of 1 and a width of AR; (2) build four inscribed circles inside the rectangle with a radius *r*; and (3) solve Equation (1) to obtain the radius *r* given a value of C. In Equation (1), $\theta_{1}=\arccos\frac{AR-2r}{2r}$ and $\theta_{2}=\arccos\frac{1-2r}{2r}$. It should be noted that Equation (1) has solutions only if AR > 0.5 and C is close to unity. Such ranges of the two descriptors can ensure that the four-particle model is a good representative for Fujian sand according to cumulative distribution curves in Figure 1. Once given the descriptors, circles with pre-assigned radii can be assembled to form desirable particle clumps that have the same size as the target particles and achieve calibration grading (Figure 1c).

(1) $$\frac{AR-\left(4-\pi \right)r^{2}-2\left[r(AR-2r)-\pi r^{2}\frac{90-\theta_{1}}{180}-\frac{1}{2}r^{2}\sin2\theta_{1}\right]-2\left[r(1-2r)-\pi r^{2}\frac{90-\theta_{2}}{180}-\frac{1}{2}r^{2}\sin2\theta_{2}\right]}{AR-\left(4-\pi \right)r^{2}}=C$$

### 2.2. Constitutive Model of Contact and Calibration

**Figure 3:**
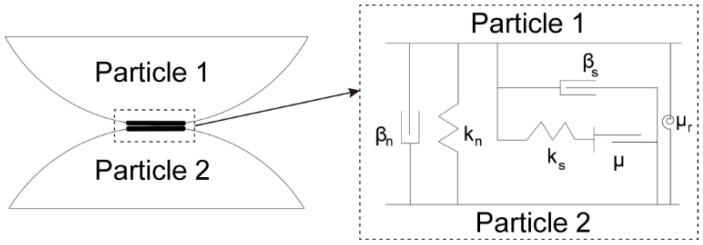
Schematic of the constitutive model of particle-particle contact.

The DEM-based particle flow code (PFC2D) [40] is selected for constructing the particle clump and the corresponding contact constitutive model. For particles inside the clump, they were bonded to each other and the particle would not be broken during the simulation. For the contact between particle clumps, we adopted the rolling resistance linear model [40,41] to reproduce the contact behavior between particles, which was extended from the classic linear model, as shown in Figure 3. The rolling resistance linear model is controlled by the linear contact stiffness (normal and shear stiffnesses *k*_n_ and *k*_s_, respectively), contact friction (friction coefficient *μ* and rolling friction coefficient *μ*_r_) and damping (normal and shear damping ratios *β*_n_ and *β*_s_, respectively). In the model, the rolling resistance moment will not further increase once it exceeds a certain threshold determined by the rolling contact friction.

**Figure 4:**
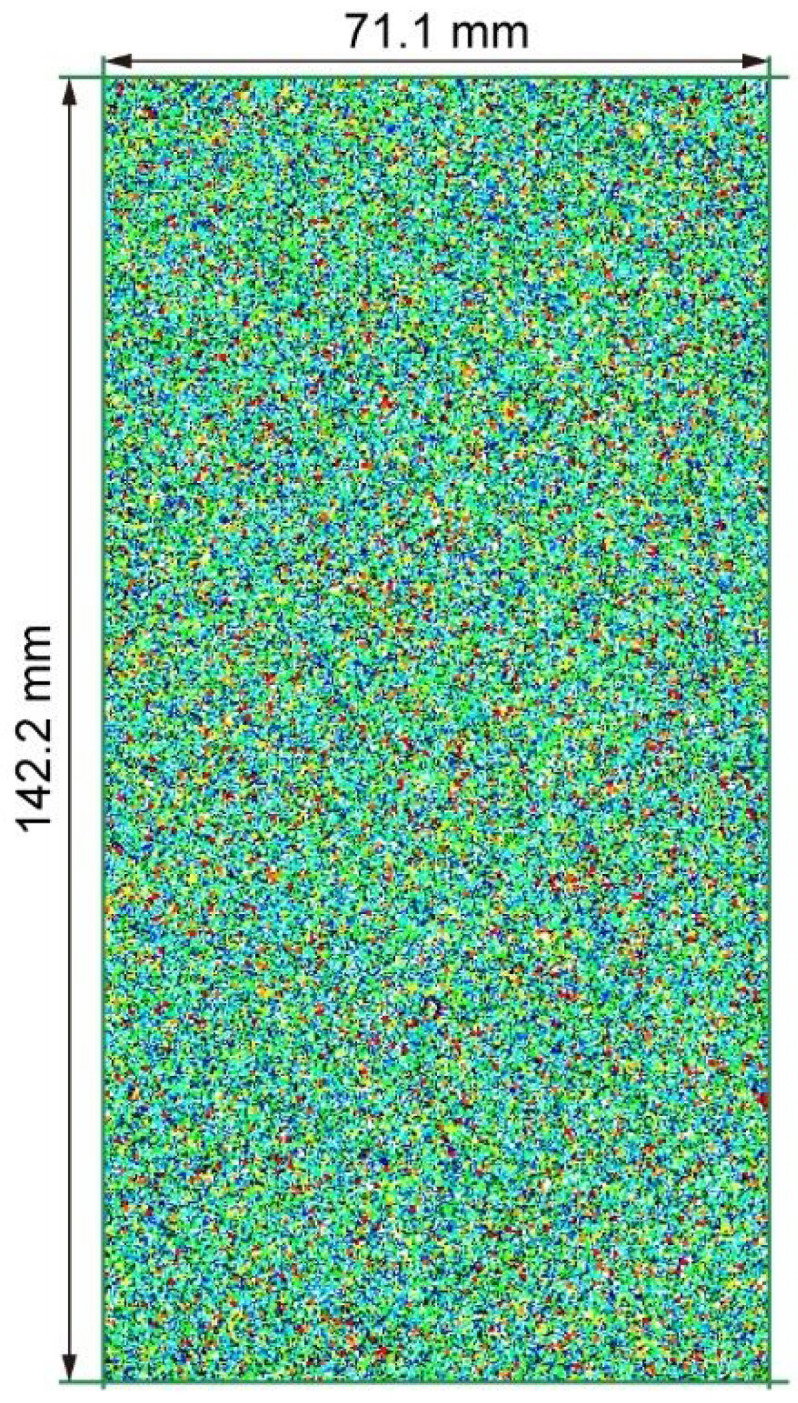
Numerical calibration model in PFC2D.

**Figure 5:**
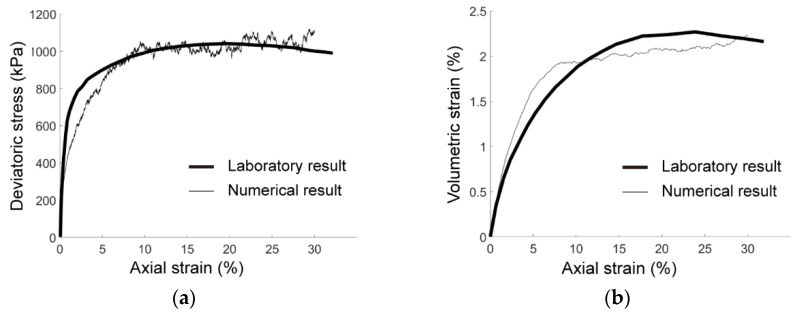
Numerical calibration results: (**a**) deviatoric stress as a function of axial strain; (**b**) volumetric strain as a function of axial strain.

The calibration model was bounded by four walls that formed a rectangular domain with a size of 142.2 mm × 71.1 mm, as shown in Figure 4. Based on the laboratory results of Fujian sand [39] shown in Figure 1, we adopted five groups of particle clumps with different descriptors, of which each had an equal proportion of the total particles, i.e., 20%. The descriptors are listed in Table 1. In the calibration model, there were 147,712 circles forming 36,928 clumps generated. The density and initial porosity of the calibration sample were set as 2640 kg/m^3^ and 0.2, respectively. Figure 5 compares the relationship curves obtained in the laboratory tests (thick lines) and numerical simulations (thin lines) under the effective confining pressure of 500 kPa, and Table 2 gives the associated values for the micro parameters of the rolling resistance linear model. In the initial stage, the simulated elastic module (slope of the curve in Figure 5a) was slightly smaller than the experimental one, while the two curves both asymptotically converged to a constant after the axial strain exceeded about 8%. Although there was some fluctuation at the stationary phase of the numerical curve that was due to the rearrangement of local structures during the compression, the peak strength of the calibration sample was close to the laboratory one. As shown in Figure 5b, the volumetric strain derived from the numerical simulation behaved similarly to the laboratory curve, of which the yielding strain was about 2%. Figure 5 shows good agreement of our calibration model compared to the laboratory tests, indicating that the parameters in Table 2 were capable of reproducing the mechanical behavior of Fujian sand.

**Figure 6:**
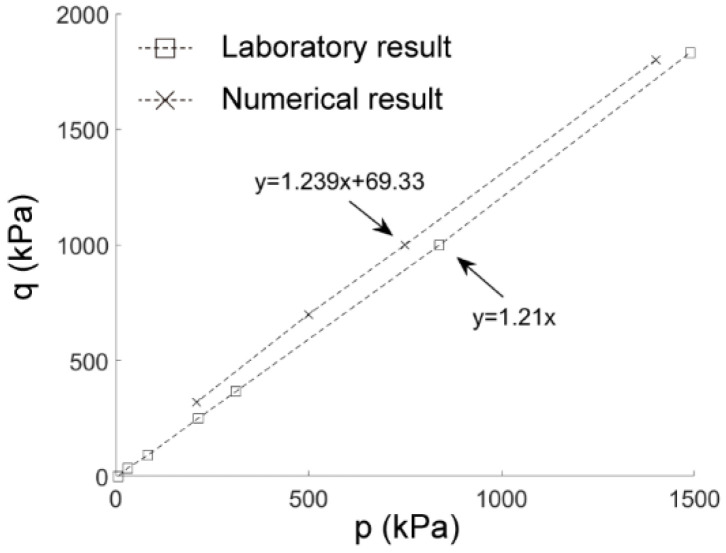
Failure envelopes for numerical calibration models and laboratory tests.

We further changed the confining pressure to obtain the failure envelope of Fujian sand as shown in Figure 6, where the slopes of the numerical and laboratory results were 1.239 and 1.21, respectively. The friction angle of the soil sample can be derived from the slope using sin*φ* = 3M/(6 + M), where M is the slope of the q–p curve. The friction angles derived from the calibration simulations and the laboratory tests were 30.90° and 30.23°, respectively, of which the error was negligible. Therefore, the calibrated parameters for the contact model listed in Table 2 were also applicative for particles under different stress conditions.

### 2.3. Setup of Circular Tunnel Excavation Model

#### 2.3.1. Model Configuration

As stated above, the calibration model with a size of 142.2 mm × 71.1 mm contained 147,712 circles forming 36,928 clumps. If using the same geometrical configuration to establish a tunnel excavation model with a size of 30 m × 30 m containing a circular tunnel of 6 m diameter, the number of circles will exceed 12 billion. Current computers could not handle such numerous particles. Therefore, we reduced the number of particles based on the principle of the centrifuge test [35,42]. In the model, we used the same particles with the parameters listed in Table 2 and set the gravitational acceleration as *N*g, where g is the original gravitational acceleration with a magnitude of 9.8 m/s^2^ and *N* is the similitude ratio (the ratio of a parameter in the numerical model to the one in the prototype model). Afterwards, the size of the numerical model was set as 1/*N*. As a consequence, the stress in the numerical model could reach an equivalent state to the realistic in situ stress state.

To balance the computational efficiency and the accuracy of the excavation model, the following assumptions were additionally set: (1) the initial stress field is generated by the gravity stress, without considering the tectonic stress field; (2) the sandy formation is dry and the effect of the ground water is neglected; (3) the tunnel is excavated within a single step and the construction duration is ignored; (4) the tunnel lining is simulated by rigid walls, of which the convergence is ignored; and (5) all the bounded walls are frictionless.

**Figure 7:**
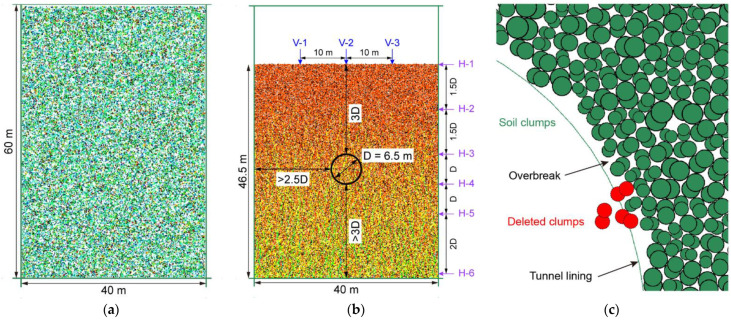
Configuration of the circular tunnel excavation: (**a**) initial model before consolidation; (**b**) consolidated model and the location of the tunnel; (**c**) tunnel lining model.

The model is bounded by four walls forming a rectangular box with a size of 150 mm × 100 mm. Herein, we used a similitude ratio of 400, i.e., the gravitational acceleration in the numerical model was 400 g. Thus, the height and width could represent a computational domain of 60 m × 40 m (Figure 7a) as the size of the numerical model is 1/400 of that of the prototype one. The establishment of the excavation model mainly consisted of two steps. In the first step of the initial model generation, the particles that were set frictionless were generated in the model box (Figure 7a), which fell down freely, consolidated and finally reached the equilibrium state under the action of gravity (Figure 7b). Afterwards, the friction coefficient was reset to 0.5. There were about 180,000 particles in total. In the following step of the tunnel excavation, the tunnel diameter (D) was set to 6.5 m and buried depth to 19.5 m, i.e., 3 times that of the diameter, as shown in Figure 7b. Since the width of the model was 40 m, i.e., larger than 5 D, the distance between the side boundary and the tunnel lining was larger than 2.5 D (Figure 7b). The height of the consolidated ground was 46.5 m, ensuring a distance between the lower boundary and the tunnel lining larger than 3 D (Figure 7b). Such a configuration could eliminate the boundary effect. The excavation was simulated by deleting the particles within the range of the tunnel and the rigid walls were synchronously installed to model the tunnel lining (Figure 7c). Afterwards, the model was executed for 30 time-steps and a single time-step (T) was set to 2 × 10^−4^ s. Finally, the ground reached a balanced stress state after particles converged towards the lining due to overbreak.

#### 2.3.2. Numerical Experimental Design

In this excavation model, we used the same micro mechanical parameters of particle contact as listed in Table 2. We examined five cases with different descriptors for particle shape, i.e., AR2C1 (AR = 0.7, C = 0.95), AR2C2 (AR = 0.7, C = 0.97), AR2C3 (AR = 0.7, C = 0.99), AR4C3 (AR = 0.85, C = 0.99) and AR6C3 (AR = 1.0, C = 0.99), among which the AR2C3 case was selected as the basic case for contrasting with other cases to investigate the influences of AR and C on the ground response due to tunnel excavation. The model was divided into 30 × 30 subdomains, where the stress tensor (*σ_xx_*, *σ_xy_*, *σ_yx_* and *σ_yy_*) was recorded during the simulation. In addition, we recorded the particle displacement inside each subdomain and derived the average displacement of the subdomain as

(2) $$z_{ij}=\frac{\sum_{c\in N_{p}}V_{c}\cdot z_{c}}{\sum_{c\in N_{p}}V_{c}},$$

where *z_ij_* is the averaged displacement of the subdomain located at the *i*th row and *j*th column, *N_p_* is the number of particle clumps inside the subdomain, *V_c_* represents the particle volume and *z_c_* is the particle displacement (*x*-direction or *y*-direction). To better demonstrate the simulation results, we further established three vertical and six horizontal measuring lines as shown in Figure 7b.

### 2.4. Microscopic Parameters

#### 2.4.1. Principal Stress

We derived the maximum and minimum principal stresses as

(3) $$\sigma_{\max,\min}=\frac{1}{2}\left(\sigma_{xx}+\sigma_{yy}\right)\pm \sqrt{{\left[\frac{1}{2}\left(\sigma_{xx}+\sigma_{yy}\right)\right]}^{2}+\sigma_{xy}^{2}},$$

where *σ_xx_*, *σ_xy_* and *σ_yy_* are the components of the stress tensor and *σ*_max_ and *σ*_min_ are maximum and minimum principal stresses, respectively. We further calculated the stress variation angle, *α*, i.e., the angle anticlockwise from the positive *x*-direction to the principal stress, as

(4) $$\alpha =\frac{1}{2}\tan^{-1}\left(\frac{-2\sigma_{xy}}{\sigma_{xx}-\sigma_{yy}}\right).$$

#### 2.4.2. Void Ratio

Inside each measuring subdomain, we defined the void ratio, *n*, as the ratio of void volume to the total volume of the subdomain. As the overlapping between particles is very small and can be negligible, the void ratio can be further derived from the particle occupation as

(5) $$n=\frac{V_{\mathrm{void}}}{V}=1-\frac{V_{\mathrm{ball}}}{V},$$

where *V*_void_ is the void volume inside the subdomain, *V*_ball_ is the occupation volume of the particles and *V* is the volume of the subdomain.

#### 2.4.3. Coordination Number

The coordination number is the average number of contacts per particle and a measure of the packing density of a granular assembly [43,44], which is defined as

(6) $$C_{n}=\frac{2N_{c}}{N_{p}},$$

where *N_c_* and *N_p_* are the numbers of contacts and particles, respectively.

## 3. Results

### 3.1. Stress Distribution

**Figure 8:**
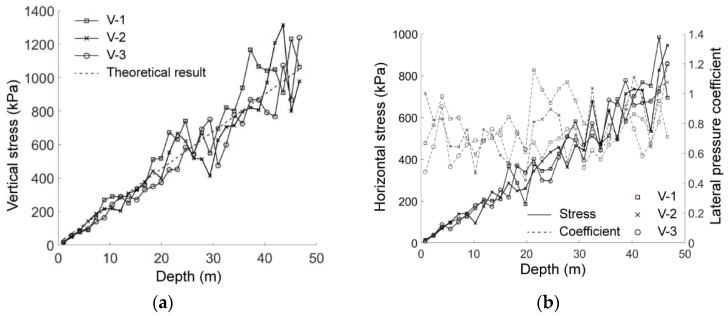
The stress state of the ground before excavation: (**a**) vertical stress, *σ_yy_*, as a function of depth; (**b**) horizontal stress, *σ_xx_*, (solid line) and lateral pressure coefficient (dashed line) as a function of depth.

Figure 8 shows the stress state as a function of the depth in the basic case of AR2C3. As shown in Figure 8a, the vertical stresses of the three vertical measuring lines all increased linearly with depth, in agreement with the trend of the theoretical result. Although the fluctuation became more violent as the depth increased, the general trend of the numerical results agreed well with the theoretical one, showing the effectiveness of the excavation model. The horizontal stress shown in Figure 8b behaved similarly to the vertical one. The lateral pressure coefficient (the ratio of horizontal stress to vertical stress) fluctuated around an average value of 0.764, within the typical range of a sandy formation.

**Figure 9:**
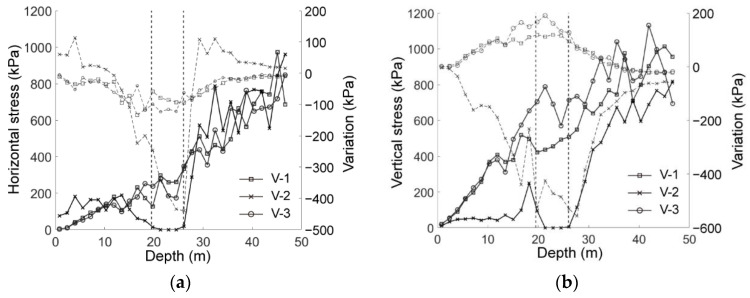
The stress state of the ground after excavation: (**a**) distribution of horizontal stress, *σ_xx_*, (solid line) and its variation (dashed line); (**b**) distribution of vertical stress, *σ_yy_,* (solid line) and its variation (dashed line). The two vertical dashed lines represented the range of the tunnel.

After the tunnel excavation, we plotted the vertical and horizontal stresses and their variations in Figure 9, where the two vertical dashed lines represent the range of the tunnel, i.e., depth from 19.5 to 26 m. It was observed that, after the tunnel excavation, the vertical stress along the measuring line of V-2 sharply reduced to around zero within the tunnel opening space whereas the stresses along V-1 and V-3 increased near the range of the tunnel space (Figure 9a). As shown in Figure 9b, the horizontal stress along V-2 reduced within the range of the tunnel while it tended to be larger than the initial state in the range of 0.5 D outward of the excavation on both sides. The horizontal stresses measured along V-1 and V-3 both reduced with a decreasing trend as the distance from the tunnel increased. The redistribution of the stress showed that the vertical stress was transferred onto both sides of the tunnel excavation and the horizontal stress increased above the excavation, forming an obvious soil arching around the tunnel.

### 3.2. Ground Deformation

**Figure 10:**
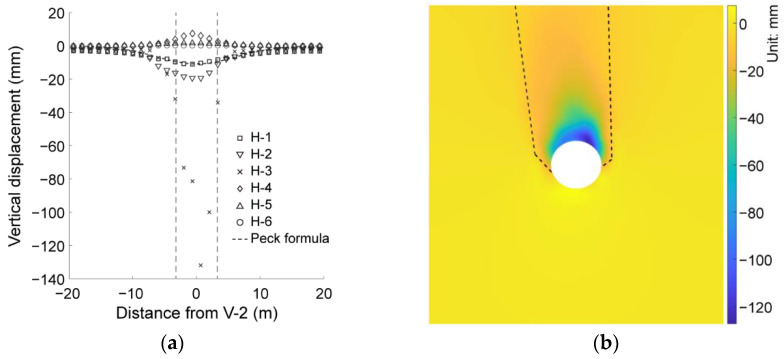
(**a**) Vertical displacement recorded along six horizontal measuring lines with a comparison with the prediction by the Peck formula. The two vertical dashed lines represent the range of the tunnel. (**b**) Distribution of the ground settlement.

Ground deformation is an important indicator reflecting the ground response due to tunnel excavation. Figure 10a illustrates the settlement recorded in each horizontal measuring line in the basic case of AR2C3, where the positive and negative of the horizontal axis indicates the left and right sides with respect to the central axis of the tunnel (i.e., the V-2 line), respectively. It can be observed that the vertical displacements above and beneath the tunnel had different trends: the displacement of lines H-1, H-2 and H-3 dropped downwards while the displacement of lines H-4, H-5 and H-6 had an upward tendency. The opposite response of the ground above and below the tunnel was due to the unloading effect of the excavation that made the surrounding soil move toward the excavation opening. The variation in the displacement of each measuring line along the horizontal axis behaved similarly, no matter if it was a settlement or an uplift movement. We then plotted the prediction result using the Peck formula in Figure 10a (dashed line), where the surface settlement (H-1) had good agreement with the Peck prediction. A more detailed distribution of the ground settlement is given in Figure 10b, where an obvious chimney-shaped area can be recognized as the excavation-influenced area. It can be also observed that the maximum displacement of each measuring line did not exactly occur at the central line of the tunnel (Figure 10a) and the excavation-influenced area did not have a symmetrical shape (Figure 10b). However, for the simulations on the ground formed by rounded particles, the ground settlement and the excavation-influenced area are almost symmetrical with respect to the central line [45,46,47,48]. Therefore, the particle shape-induced heterogeneity may have a negligible effect on the excavation-induced responses.

### 3.3. Interaction between Ground and Tunnel Lining

**Figure 11:**
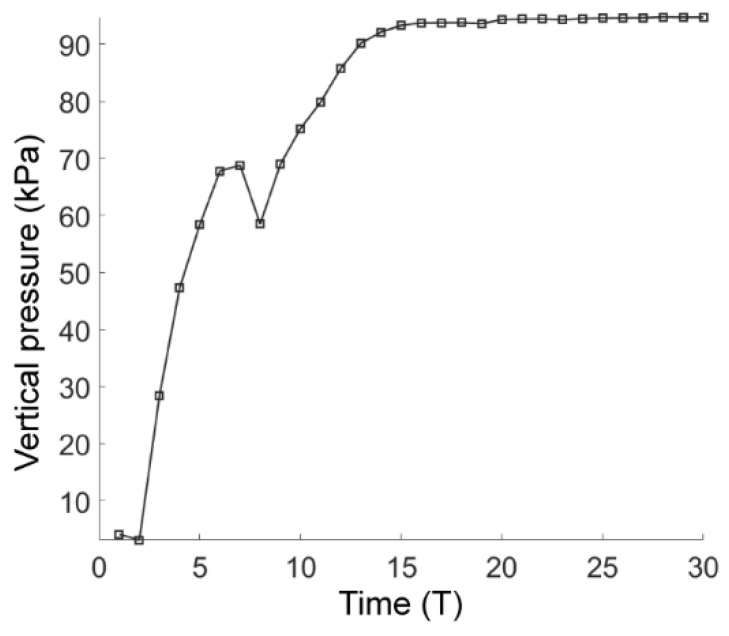
Average vertical pressure on the tunnel lining as a function of computational time.

During the simulation after excavation, we recorded the normal contacting forces between particles and the lining element, which was the representative of the pressure on the tunnel lining from the surrounding sandy formation. It should be noted that the tunnel lining was in an equilibrium state under the soil pressure from the overburden and underlying ground. Thus, we derived the average vertical pressure, *σ_v_*, from the overburden soil as

(7) $$\sigma_{v}=\frac{\int_{0<\beta <\pi }\sigma_{c}\sin\beta \frac{D}{2}d\beta }{D}=\frac{1}{2}\int_{0<\beta <\pi }\sigma_{c}\sin\beta d\beta ,$$

where *β* is the angle anticlockwise from the positive *x*-direction to the line formed by the contact point and the tunnel center and *σ_c_* is the normal contact stress. The vertical pressure on the tunnel lining in the basic case of AR2C3 is plotted in Figure 11, where the soil pressure increased rapidly soon after the excavation and gradually converged to a steady value of 94 kPa after the computational time reached 16 T.

### 3.4. Microscopic Characteristics of Ground Response

**Figure 12:**
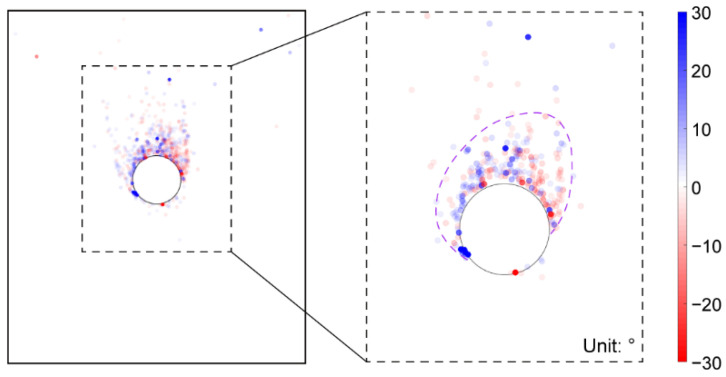
Particle rotation after the tunnel excavation in the basic case of AR2C3.

Figure 12 illustrates the particles’ rotational displacements after the tunnel excavation, where the positive value represents the counterclockwise angle and vice versa. The figure showed that most of the particles with large rotation angle were concentrated near the top of the excavated tunnel. The farther away from the tunnel, the smaller the rotation angle of the particles. It was implied that the excavation-induced particle rotation was only restricted in a very limited area around the tunnel, i.e., the farther away from the tunnel, the smaller the excavation influence. It can also be observed that the particles on the right side above the tunnel mainly rotated clockwise, while the particles on the upper left side mainly rotated counterclockwise. Incorporated with the position of the tunnel, the microscopic performance of the upper soil after the tunnel excavation was to roll downward along the lining surface. As shown in the inset of Figure 12, the rotated particles were mainly located within an anomalous area, implying that the particle shape-induced heterogeneity has a significant role on the particle rotational deformation after the tunnel excavation.

**Figure 13:**
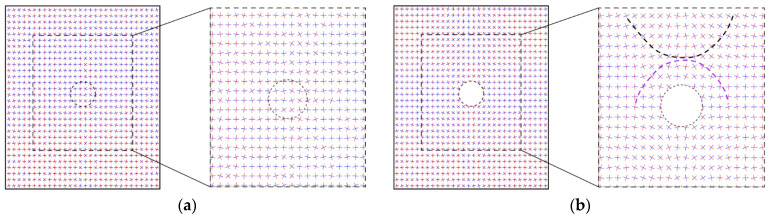
Directions of the principal stresses (**a**) before and (**b**) after the tunnel excavation. The red and blue segments indicate the maximum and minimum principal stresses, respectively.

**Figure 14:**
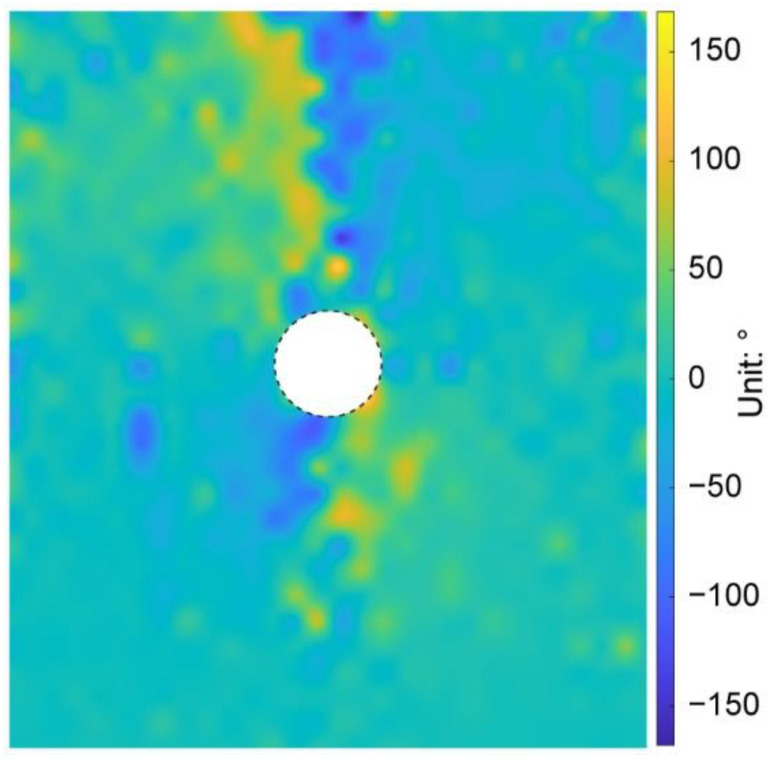
Distribution of the stress variation angle after the tunnel excavation in the basic case of AR2C3.

We then plotted the directions of the principal stresses before and after the tunnel excavation in Figure 13, where the red and blue segments indicate the maximum and minimum principal stresses, respectively. As shown in Figure 13a, the direction of the maximum principal stress mainly focused on the vertical before the tunnel excavation. However, due to the heterogeneous ground induced by the effect of particle shape, there were still some positions where the stress directions had a slight deflection. After the tunnel excavation, if the maximum principal stresses above the tunnel are connected to each other, a bowl-shaped curve can be formed (black curve in Figure 13b), showing that the rotation of the maximum principal stress mainly occurred just above the tunnel. However, the maximum principal stresses above the tunnel shoulders did not rotate a lot. Such a distribution of the stress rotation can form an effective soil arching above the tunnel (purple curve in Figure 13b). We further demonstrate the distribution of the stress variation angle derived by Equation (4) in Figure 14, where the maximum principal stress on the upper right and lower left of the tunnel rotated clockwise while the one on the lower right and upper left rotated counterclockwise. The rotation of the maximum principal stress above the tunnel was consistent with the particle rotation shown in Figure 12. The asymmetry phenomena were obviously observed in the bowl-shaped rotation curve of the maximum principal stress and the soil arching in Figure 13, and in the distribution of the stress variation angle in Figure 14, implying that the ground heterogeneity caused by the particle shape effect has a significant role in the ground response after tunnel excavation.

**Figure 15:**
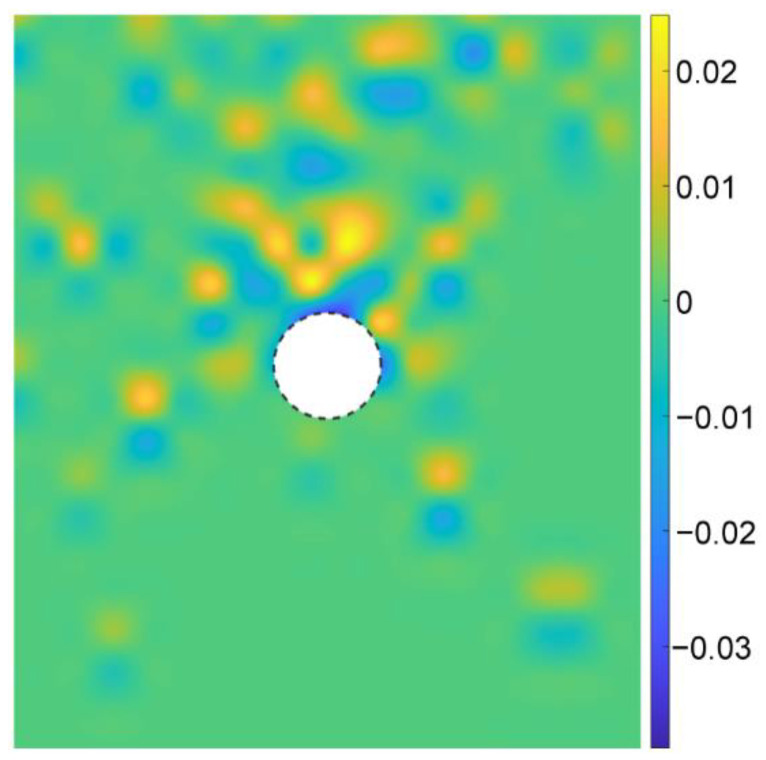
Variation in the void ratio after the tunnel excavation in the basic case of AR2C3. The negative value indicates the excavation-induced reduction of void ratio.

Figure 15 shows the variation in the void ratio between the initial state and the stationary state after the tunnel excavation in the basic case of AR2C3, where the negative value indicated the reduced void ratio induced by excavation. The void ratio under the tunnel did not change a lot whereas the value near the tunnel became smaller, which was caused by the filling of the overbreak due to tunnel excavation. There was a limited area with a large variation in the void ratio over the low-value area above the tunnel, indicating the emergence of a local collapse. Particles in this area fell and filled up the gaps between the ground and the tunnel lining. In the farther area above this collapsed region, the void ratio had little variation, implying that the formed soil arching (Figure 13b) supported the ground and prevented further failure. An obvious asymmetry was also observed in the distribution of the variation in the void ratio.

**Figure 16:**
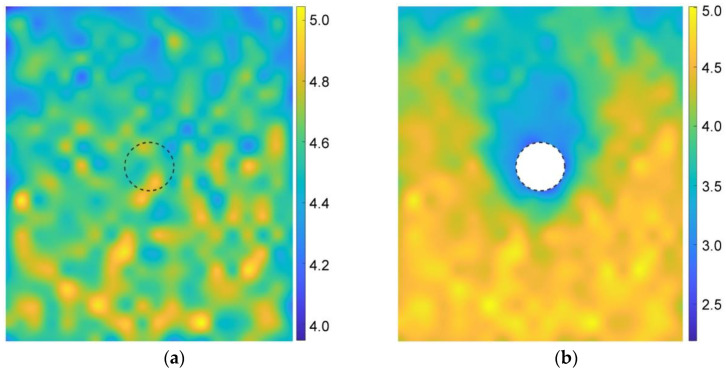
Distribution of coordination numbers of the sandy ground (**a**) before and (**b**) after the tunnel excavation.

According to Equation (6), Figure 16 gives the coordination numbers before and after the tunnel excavation for the basic case of AR2C3. In the initial stress state (Figure 16a), the magnitude of the coordination number generally had a positive relationship with the depth, i.e., the higher the stress, the larger the coordination number. After excavating the tunnel, the coordination number remained unchanged with a magnitude of around 4.5. However, the coordination number around the tunnel reduced to about 3.0. The region where the coordination number reduced was consistent with the area where particles rotated, as well as the region of excavation-influenced settlement, inducing a very loose space above the tunnel.

**Figure 17:**
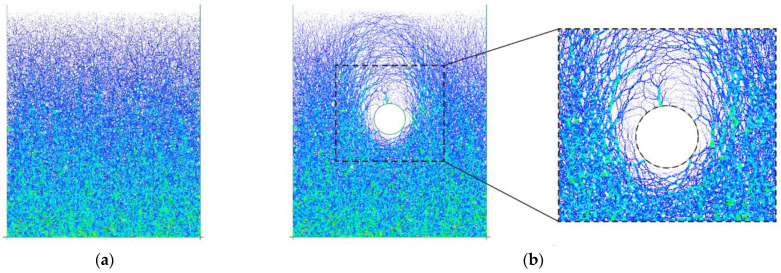
Force chains of the sandy ground (**a**) in the initial state and (**b**) after the tunnel excavation.

**Figure 18:**
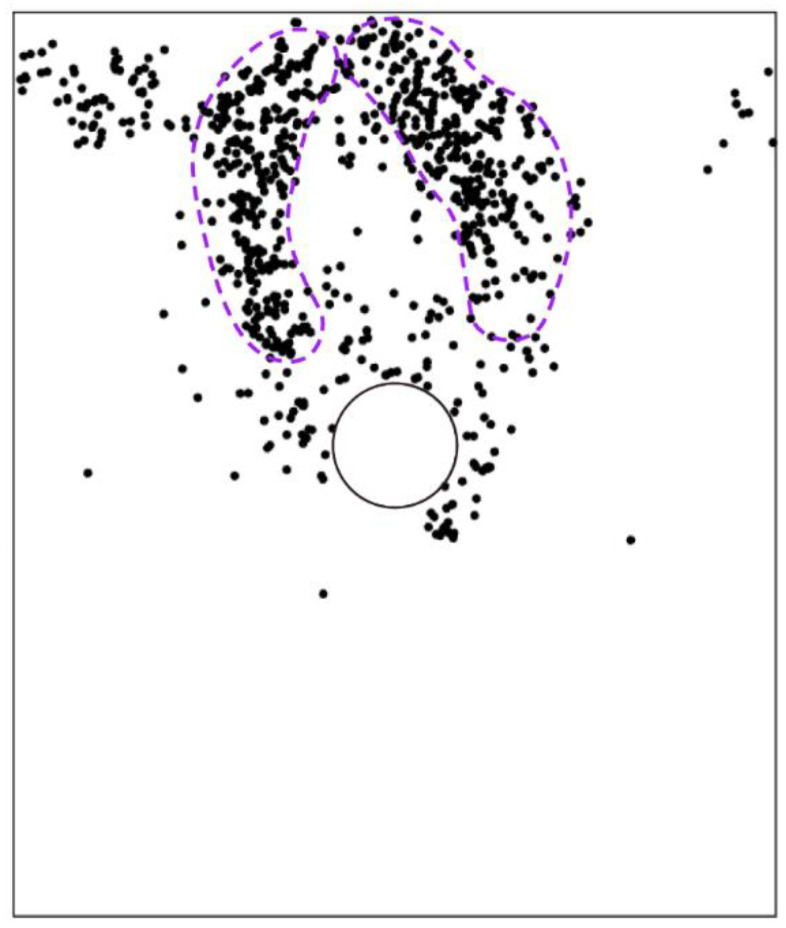
Distribution of shear-slip contacts. The purple curves indicate two shear bands.

For the basic case of AR2C3, Figure 17 shows the force chains generated in the initial state and after the tunnel excavation, where the thicker lines indicate larger contact forces. In the initial stress state, the contact forces became larger as the depth went deeper, which agreed well with the distribution of the coordination number shown in Figure 16a. After the excavation, the force chains around the tunnel tended to be thinner and sparser, i.e., a loose area was formed around the tunnel due to the excavation-induced unloading. As shown in the inset of Figure 17b, the force chains formed a soil arching above the tunnel that supported the overburden ground, reducing the soil pressure acting on the lining. The force chains far away from the tunnel remained unchanged after the excavation. We further plotted the distribution of contacts where the shear slip emerged in Figure 18. Two obvious shear bands could be identified (purple curves in Figure 18), which were consistent with the boundaries of the excavation-induced settlement (Figure 10b), indicating that particles dislocated in these areas when the ground settlement occurred. By comparing Figure 12 and Figure 18, it can be observed that no obvious correlation was recognized between the shear slip and the particle rotation as they were two different motion patterns. Particles in the shear bands rarely rotated, i.e., the shear slip between particle was a type of translation. Meanwhile, in the loose zone above the tunnel, most particles rotated clockwise or counterclockwise, whereas only a small quantity of particles slipped. It should be noted that, similar to the ground settlement, the microscopic characterization of the ground response due to tunnel excavation also had an obvious asymmetry caused by the effect of particle shape. In the following section, we will discuss how the particle shape descriptors affect the ground response.

## 4. Discussion

### 4.1. Effect of Aspect Ratio

**Figure 19:**
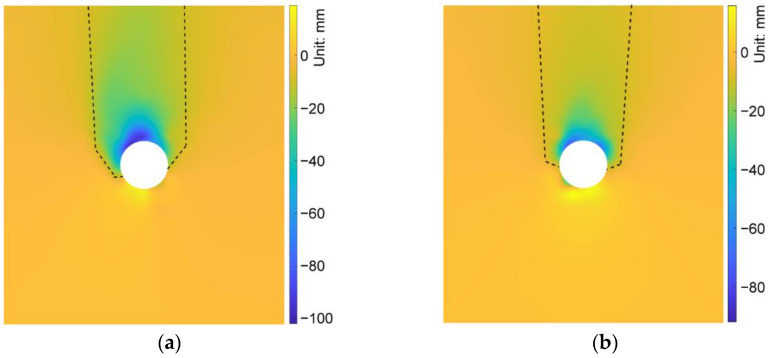
Distribution of the ground settlement in the cases of (**a**) AR4C3 and (**b**) AR6C3.

**Figure 20:**
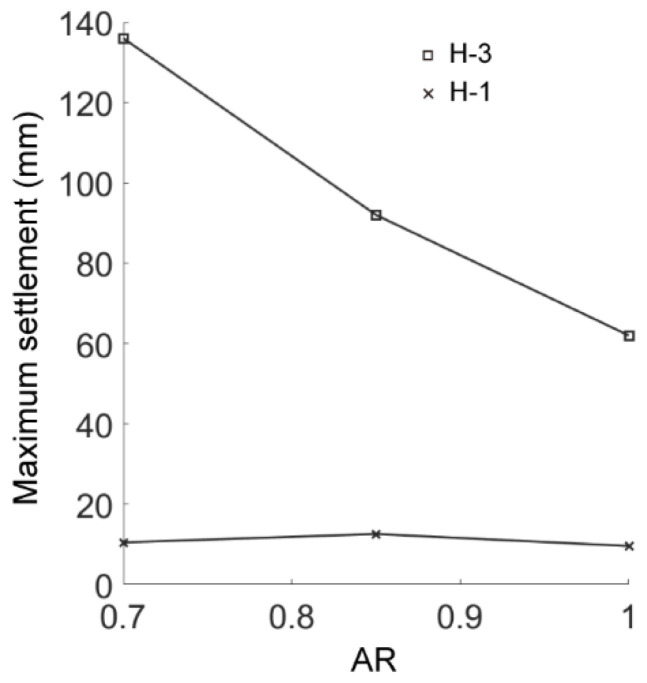
Maximum settlements of measuring lines H-1 and H-3 as a function of AR.

We compared the simulation results in the cases of AR2C3, AR4C3 and AR6C3 to investigate the influence of AR of sandy particles on the excavation-induced ground response. Figure 19 demonstrates the distribution of the ground settlement of AR4C3 and AR6C3. Incorporating Figure 10b, the increased AR would enlarge the excavation-influenced region, but the boundaries of the chimney-shaped settlement were more difficult to recognize. As shown in Figure 20, a larger value of AR would reduce the maximum settlement above the tunnel vault whereas AR did not significantly contribute to the ground surface settlement. It should also be noted that the asymmetry still existed when AR = 0.85, whereas the settlement in the case of AR = 1.0 was generally symmetrical, implying that the emergence of elongated particles was the primary inducement of the asymmetrical ground settlement.

**Figure 21:**
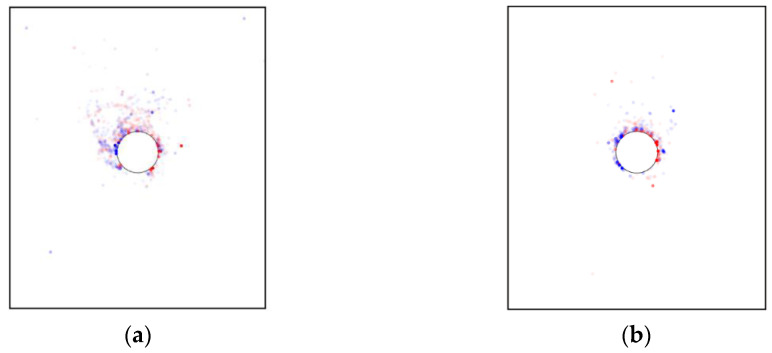
Particle rotation after the tunnel excavation in the cases of (**a**) AR4C3 and (**b**) AR6C3. Refer to Figure 12 for the legend.

**Figure 22:**
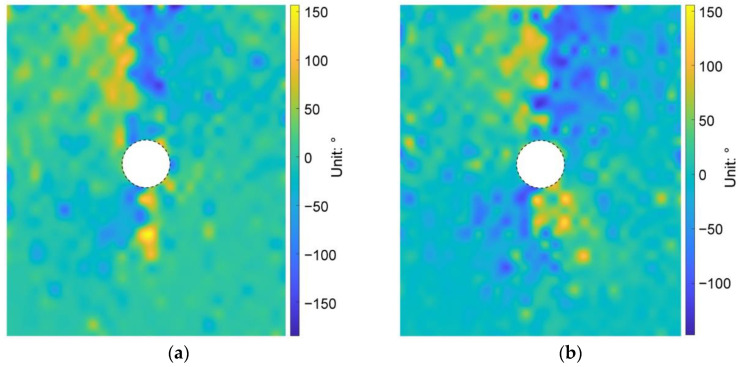
Stress variation angle after the tunnel excavation in the cases of (**a**) AR4C3 and (**b**) AR6C3.

**Figure 23:**
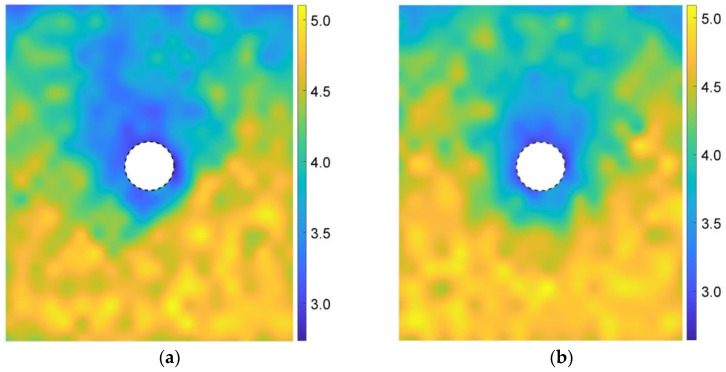
Distribution of coordination numbers of the ground after the tunnel excavation in the cases of (**a**) AR4C3 and (**b**) AR6C3.

We further explored the influence of AR on the microscopic characteristics of the ground response. Figure 21 illustrates the particle rotation after the tunnel excavation for the cases of AR4C3 and AR6C3. Similar to the case of AR2C3 (Figure 12), the particles on the right side above the tunnel mainly rotated clockwise, whereas the particles on the upper left side mainly rotated counterclockwise. Compared with Figure 12, the range where particles rotated reduced as AR increased, implying that the larger AR was, i.e., the particle were more rounded, the less likely a particle rotation was to drive the surrounding particles to rotate. As for the stress variation angle (Figure 14 and Figure 22) and the coordination number (Figure 16b and Figure 23), the variation in AR did not alter the general distribution characteristics. However, the range of the excavation-induced variation in these two descriptors became wider when AR increased, which was consistent with the ground settlement. In addition, the ground responses with respect to these microscopic viewpoints in the case of AR = 1.0 behaved less asymmetrically than the other two cases, indicating that the asymmetrical ground response was mainly caused by the prolate particles rather than the rounded ones.

**Figure 24:**
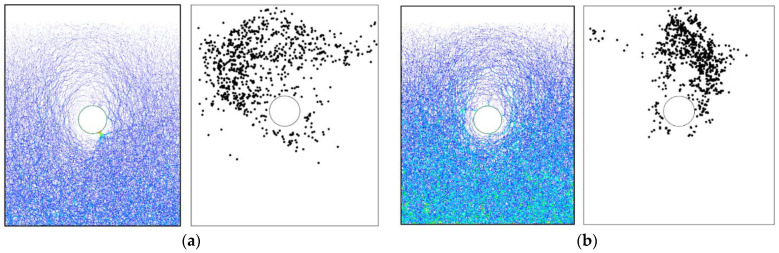
Force chains of the sandy ground and shear-slip contact distribution after excavation in the cases of (**a**) AR4C3 and (**b**) AR6C3.

**Figure 25:**
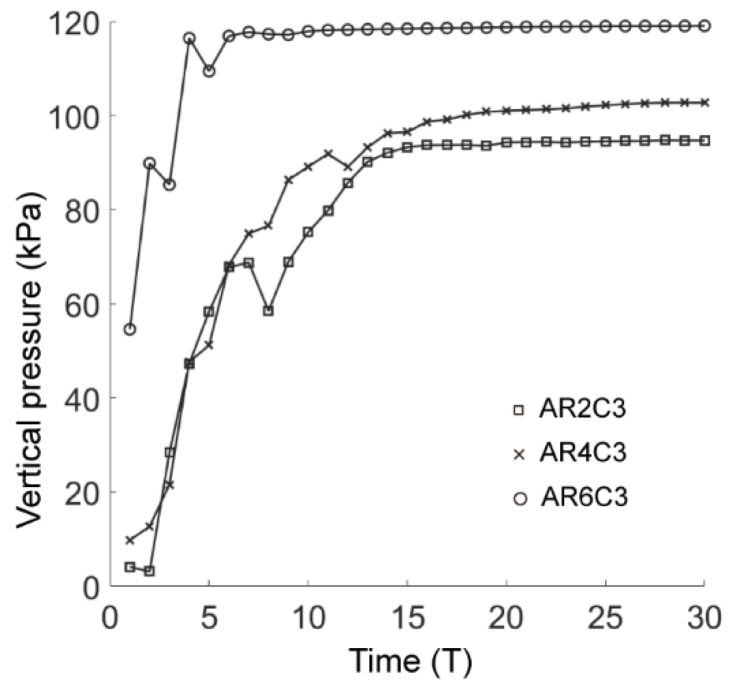
Average vertical pressure on the tunnel lining as a function of computational time in all three cases of C = 0.99.

Figure 24 shows the force chains and the shear-slip contacts for the cases of AR4C3 and AR6C3. Similar to the case of AR2C3, the force chains around the tunnel formed a series of chain arching, generating a stable soil arching to support the overburden pressure. When AR was small, i.e., 0.7, the soil arching structure was fully developed and the shear-slip contacts were evenly distributed along the soil arching. For larger values of AR, the shear-slip zones deviated to the left or right. The shear bands developed on the one side above the tunnel while the other side merely had a few shear-slip contacts. As stated above, the soil arching was beneficial for reducing the soil pressure on the tunnel lining, we thus plotted the average vertical pressure on the tunnel lining as a function of computational time for all the three cases in Figure 25. The soil pressure vertically on the lining converged earlier to a steady state in the sandy soil with more rounded particles. The magnitude of the soil pressure had a positive relationship to AR, i.e., the soil arching in the sandy ground formed by more rounded particles bore less overburden loading. It can be learnt that elongated particles are beneficial for the generation of the soil arching with stronger bearing capacity.

### 4.2. Effect of Convexity

**Figure 26:**
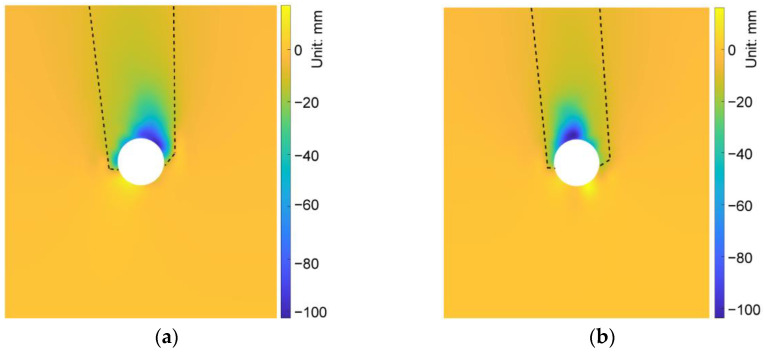
Distribution of the ground settlement in the cases of (**a**) AR2C1 and (**b**) AR2C2.

**Figure 27:**
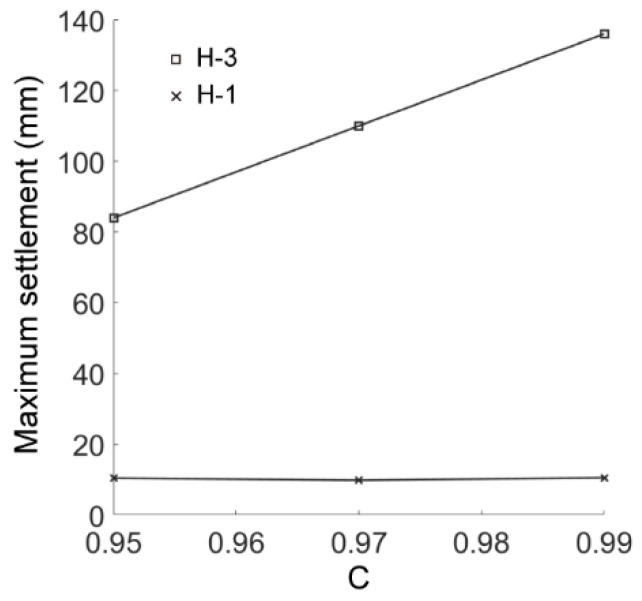
Maximum settlement of measuring lines H-1 and H-3 as a function of Convexity.

We then changed the values of C but preserved a constant AR of 0.7 to obtain the results for the cases of AR2C1 and AR2C2. Figure 26 shows the distribution of the ground settlement for the two cases, where the shapes and ranges of the settlement were similar to those of case AR2C3 (Figure 10b). Thus, the convexity of particles has no significant effect on the excavation-influenced region of ground settlement. As shown in Figure 27, the maximum surface settlement (H-1) was not affected by the varying C while the maximum settlement right above the tunnel (H-3) had a positive linear relationship to C. The asymmetry of the settlement chimney was preserved similarly in all three cases, i.e., the asymmetry was mainly the inducement by elongation rather than the convexity of particles.

**Figure 28:**
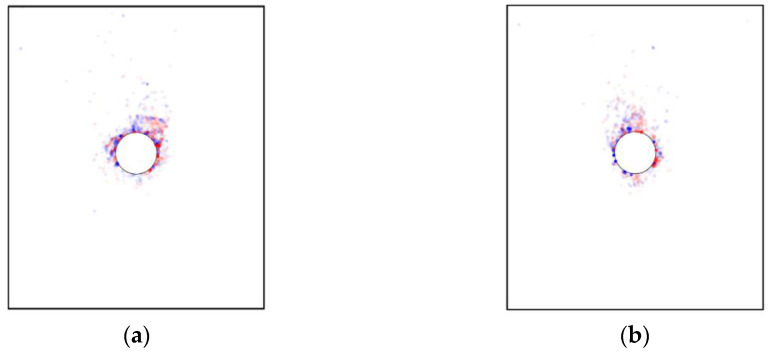
Particle rotation after the tunnel excavation in the cases of (**a**) AR2C1 and (**b**) AR2C2. Refer to Figure 12 for the legend.

**Figure 29:**
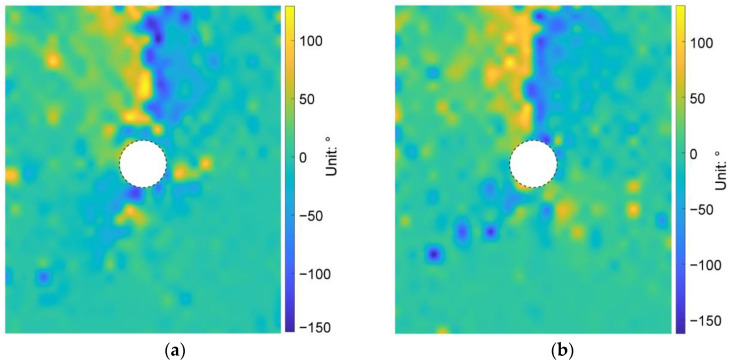
Stress variation angle after the tunnel excavation in the cases of (**a**) AR2C1 and (**b**) AR2C2.

**Figure 30:**
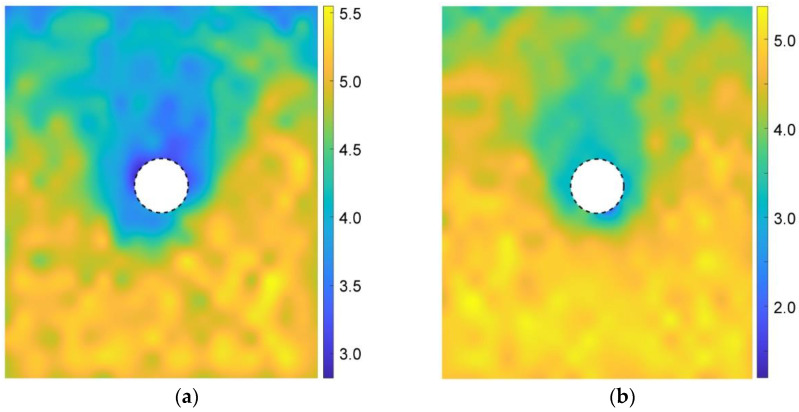
Distribution of coordination numbers of the ground after the tunnel excavation in the cases of (**a**) AR2C1 and (**b**) AR2C2.

As shown in Figure 28, similar to the case of AR2C3, the particle rotation happened to a large region and the variation in C values did not change the range. The difference mainly focused on the more obvious asymmetry and skewness as C increased. In addition, the convexity seemed to have no significant influence on the excavation-influenced region determined by the stress variation angle (Figure 29) and the coordination number (Figure 30). The asymmetrical features of the distributions for these three micro descriptors remained unambiguous for different values of C, which was consistent with the observation above.

**Figure 31:**
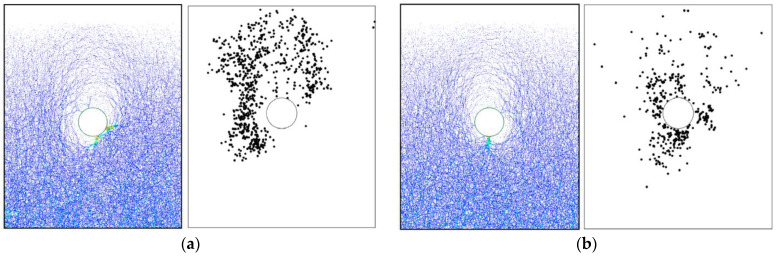
Force chains of the sandy ground and shear-slip contact distribution after excavation in the cases of (**a**) AR2C1 and (**b**) AR2C2.

**Figure 32:**
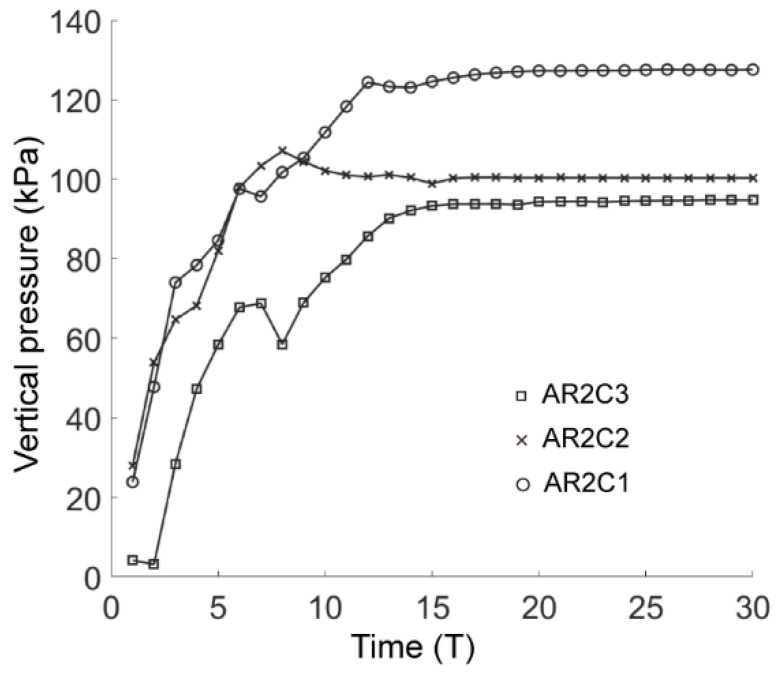
Average vertical pressure on the tunnel lining as a function of computational time in all three cases of AR = 0.7.

Figure 31 demonstrates the force chains and the corresponding shear-slip contacts in the cases of AR2C1 and AR2C2. Similar to the case of AR2C3, the force chains around the tunnel formed a stable soil arching to support the overburden pressure. However, the distribution of shear-slip contact showed that AR2C2 behaved differently from the other two as there was no distinguishable shear bands generated above the tunnel. We further compared the temporal feature of the average vertical pressure on the tunnel lining in Figure 32. The emergence time of steady state seemed to be irrelevant to the varying C. However, the pressure became smaller as C increased, indicating that the particles with a more concave shape may generate a weaker soil arching, leaving more overburden pressure on the tunnel lining.

## 5. Conclusions

In this paper, a series of 2D DEM simulations were conducted on the basis of the principle of the centrifuge test to investigate the role of particle shape on the ground response induced by the excavation of a common circular tunnel in sandy formations. The aspect ratio (AR) and convexity (C) were selected as the descriptors for the particle shape and the particle clump was generated using the four-particle model to achieve the desirable values of descriptors, which had been thoroughly calibrated. We analyzed the stress states before and after the excavation, the excavation-induced ground settlement and the soil pressure on the tunnel lining from a macro perspective. Afterwards, the microscopic characteristics of the ground response were demonstrated in detail, including the particle rotation, stress variation angle, void ratio, coordination number, force chains and shear-slip contact distribution. Finally, the influences of AR and C on the ground response in sandy soil were discussed with respect to the micro and macro perspectives. The main conclusions are drawn as follows.

(1) The asymmetry is ubiquitously observed in the micro and macro representations in all cases except for the case of AR = 1, indicating that the asymmetrical ground response in sandy formation is mainly caused by the elongated particles rather than the rounded ones.

(2) Reducing AR or increasing C will enlarge the ground settlement above the tunnel vault, i.e., the sandy ground deformation around the tunnel is more sensitive to the particles with more irregular shapes. However, the surface settlement and the excavation-influenced settlement range are barely influenced by the particle shape.

(3) The magnitude of the soil pressure on the tunnel lining has a positive relationship with AR but a negative relationship with C, i.e., the soil arching induced by tunnel excavation in the sandy ground formed by more rounded particles has a worse ability to support the overburden loading. We can learn that elongated particles are beneficial for the generation of soil arching with stronger bearing capacity.

## Figures and Tables

**Table 1:** Descriptors for particle clumps in the numerical calibration model.

AR	C	*r*
0.59	0.92	0.265
0.68	0.94	0.27
0.74	0.96	0.285
0.80	0.98	0.32
0.87	0.992	0.37

**Table 2:** Micro parameters for the rolling resistance linear model.

Parameters	Value	Unit
Normal stiffness	2 × 10^9^	Pa
Shear stiffness	1.333 × 10^9^	Pa
Friction coefficient	0.5	-
Rolling friction coefficient	0.5	-
Damping	0.5	-

## Data Availability

Not applicable.

## References

[1] Cho G.-C., Dodds J., Santamarina J.C. (2006). Particle Shape Effects on Packing Density, Stiffness, and Strength: Natural and Crushed Sands. J. Geotech. Geoenviron. Eng..

[2] Jerves A.X., Kawamoto R.Y., Andrade J.E. (2016). Effects of Grain Morphology on Critical State: A Computational Analysis. Acta Geotech..

[3] Kawamoto R., Andò E., Viggiani G., Andrade J.E. (2018). All You Need Is Shape: Predicting Shear Banding in Sand with LS-DEM. J. Mech. Phys. Solids.

[4] Altuhafi F.N., Coop M.R., Georgiannou V.N. (2016). Effect of Particle Shape on the Mechanical Behavior of Natural Sands. J. Geotech. Geoenviron. Eng..

[5] de Bono J.P., McDowell G.R. (2015). An Insight into the Yielding and Normal Compression of Sand with Irregularly-Shaped Particles Using DEM. Powder Technol..

[6] de Bono J.P., McDowell G.R. (2016). Investigating the Effects of Particle Shape on Normal Compression and Overconsolidation Using DEM. Granul. Matter.

[7] Keramatikerman M., Chegenizadeh A. (2017). Effect of Particle Shape on Monotonic Liquefaction: Natural and Crushed Sand. Exp. Mech..

[8] Ma G., Chen Y., Yao F., Zhou W., Wang Q. (2019). Evolution of Particle Size and Shape towards a Steady State: Insights from FDEM Simulations of Crushable Granular Materials. Comput. Geotech..

[9] Lai Z., Chen Q., Huang L. (2020). Fourier Series-Based Discrete Element Method for Computational Mechanics of Irregular-Shaped Particles. Comput. Methods Appl. Mech. Eng..

[10] Nguyen H.B.K., Rahman M.M., Fourie A.B. (2021). How Particle Shape Affects the Critical State, Triggering of Instability and Dilatancy of Granular Materials-Results from a DEM Study. Geotechnique.

[11] Fang C., Gong J., Nie Z., Li B., Li X. (2021). DEM Study on the Microscale and Macroscale Shear Behaviours of Granular Materials with Breakable and Irregularly Shaped Particles. Comput. Geotech..

[12] Pan T., Tutumluer E., Anochie-Boateng J. (2006). Aggregate Morphology Affecting Resilient Behavior of Unbound Granular Materials. Transp. Res. Rec. J. Transp. Res. Board.

[13] Igwe O., Sassa K., Wang F. (2007). The Influence of Grading on the Shear Strength of Loose Sands in Stress-Controlled Ring Shear Tests. Landslides.

[14] Tutumluer E., Pan T. (2008). Aggregate Morphology Affecting Strength and Permanent Deformation Behavior of Unbound Aggregate Materials. J. Mater. Civ. Eng..

[15] Tsomokos A., Georgiannou V.N. (2010). Effect of Grain Shape and Angularity on the Undrained Response of Fine Sands. Can. Geotech. J..

[16] Xiao Y., Liu H., Chen Y., Jiang J. (2014). Strength and Deformation of Rockfill Material Based on Large-Scale Triaxial Compression Tests. II: Influence of Particle Breakage. J. Geotech. Geoenviron. Eng..

[17] Yang J., Luo X.D. (2018). The Critical State Friction Angle of Granular Materials: Does It Depend on Grading?. Acta Geotech..

[18] Yang J., Wei L.M. (2012). Collapse of Loose Sand with the Addition of Fines: The Role of Particle Shape. Geotechnique.

[19] Wei L.M., Yang J. (2014). On the Role of Grain Shape in Static Liquefaction of Sand–FInes Mixtures. Geotechnique.

[20] Lei H., Chen Z., Kang X. (2022). Examination of Particle Shape on the Shear Behaviours of Granules Using 3D Printed Soil. Eur. J. Environ. Civ. Eng..

[21] Abedi S., Mirghasemi A.A. (2011). Particle Shape Consideration in Numerical Simulation of Assemblies of Irregularly Shaped Particles. Particuology.

[22] Asadi R., Mirghasemi A.A. (2018). Numerical Investigation of Particle Shape on Mechanical Behaviour of Unsaturated Granular Soils Using Elliptical Particles. Adv. Powder Technol..

[23] Xu M.Q., Guo N., Yang Z.X. (2021). Particle Shape Effects on the Shear Behaviors of Granular Assemblies: Irregularity and Elongation. Granul. Matter.

[24] Tong L., Wang Y.H. (2015). DEM Simulations of Shear Modulus and Damping Ratio of Sand with Emphasis on the Effects of Particle Number, Particle Shape, and Aging. Acta Geotech..

[25] Lu Z., Yao A., Su A., Ren X., Liu Q., Dong S. (2019). Re-Recognizing the Impact of Particle Shape on Physical and Mechanical Properties of Sandy Soils: A Numerical Study. Eng. Geol..

[26] Tsigginos C., Zeghal M. (2019). A Micromechanical Analysis of the Effects of Particle Shape and Contact Law on the Low-Strain Stiffness of Granular Soils. Soil Dyn. Earthq. Eng..

[27] Nie J.Y., Shi X.S., Cui Y.F., Yang Z.Y. (2022). Numerical Evaluation of Particle Shape Effect on Small Strain Properties of Granular Soils. Eng. Geol..

[28] Zhu Y., Gong J., Nie Z. (2021). Shear Behaviours of Cohesionless Mixed Soils Using the DEM: The Influence of Coarse Particle Shape. Particuology.

[29] Pinto F., Whittle A.J. (2014). Ground Movements Due to Shallow Tunnels in Soft Ground. I: Analytical Solutions. J. Geotech. Geoenviron. Eng..

[30] Pinto F., Zymnis D.M., Whittle A.J. (2014). Ground Movements Due to Shallow Tunnels in Soft Ground. II: Analytical Interpretation and Prediction. J. Geotech. Geoenviron. Eng..

[31] Mair R.J., Taylor R.N., Bracegirdle A. (1993). Subsurface Settlement Profiles above Tunnels in Clays. Géotechnique.

[32] Marshall A.M., Farrell R., Klar A., Mair R. (2012). Tunnels in Sands: The Effect of Size, Depth and Volume Loss on Greenfield Displacements. Géotechnique.

[33] Shao L., Zhou X., Zeng H. (2016). Comparison of Soil Pressure Calculating Methods Based on Terzaghi Model in Different Standards. Open Civ. Eng. J..

[34] Zhang H., Zhang P., Zhou W., Dong S., Ma B. (2016). A New Model to Predict Soil Pressure Acting on Deep Burial Jacked Pipes. Tunn. Undergr. Sp. Technol..

[35] Zhang Z.X., Hu X.Y., Scott K.D. (2011). A Discrete Numerical Approach for Modeling Face Stability in Slurry Shield Tunnelling in Soft Soils. Comput. Geotech..

[36] Yin Z.-Y., Wang P., Zhang F. (2020). Effect of Particle Shape on the Progressive Failure of Shield Tunnel Face in Granular Soils by Coupled FDM-DEM Method. Tunn. Undergr. Sp. Technol..

[37] Chen R.P., Liu Q.W., Wu H.N., Wang H.L., Meng F.Y. (2020). Effect of Particle Shape on the Development of 2D Soil Arching. Comput. Geotech..

[38] Ali U., Otsubo M., Ebizuka H., Kuwano R. (2020). Particle-Scale Insight into Soil Arching under Trapdoor Condition. Soils Found..

[39] Yang J., Luo X.D. (2015). Exploring the Relationship between Critical State and Particle Shape for Granular Materials. J. Mech. Phys. Solids.

[40] PFC. http://docs.itascacg.com/pfc600/pfc/docproject/source/manual/pfc_model_components/pfc_model_components.html?node1197.

[41] Zhu Y., Nie Z., Gong J. (2020). Influence of the Rolling-Resistance-Based Shape of Coarse Particles on the Shear Responses of Granular Mixtures. Particuology.

[42] Nomoto T., Imamura S., Hagiwara T., Kusakabe O., Fujii N. (1999). Shield Tunnel Construction in Centrifuge. J. Geotech. Geoenviron. Eng..

[43] Huang X., O’sullivan C., Hanley K.J., Kwok C.Y. (2014). Discrete-Element Method Analysis of the State Parameter. Géotechnique.

[44] Huang X., Hanley K.J., O’Sullivan C., Kwok C.Y. (2014). Exploring the Influence of Interparticle Friction on Critical State Behaviour Using DEM. Int. J. Numer. Anal. Methods Geomech..

[45] Bym T., Marketos G., Burland J.B., O’Sullivan C. (2013). Use of a Two-Dimensional Discrete-Element Line-Sink Model to Gain Insight into Tunnelling-Induced Deformations. Geotechnique.

[46] Hu X., He C., Lai X., Walton G., Fu W., Fang Y. (2020). A DEM-Based Study of the Disturbance in Dry Sandy Ground Caused by EPB Shield Tunneling. Tunn. Undergr. Sp. Technol..

[47] Hu X., Fu W., Wu S., Fang Y., Wang J., He C. (2021). Numerical Study on the Tunnel Stability in Granular Soil Using DEM Virtual Air Bag Model. Acta Geotech..

[48] Qin Y., Lai J., Gao G., Yang T., Zan W., Feng Z., Liu T. (2022). Failure Analysis and Countermeasures of a Tunnel Constructed in Loose Granular Stratum by Shallow Tunnelling Method. Eng. Fail. Anal..

